# Medical Society of the State of Tennessee

**Published:** 1886-01-20

**Authors:** 


MEDICAL SOCIETY OF THE STATE OF TENNESSEE.—Thomas L. Maddin, M. D., President, Nashville; Deering J. Roberts, M. D., Treasurer, Nashville; C. C. Fite, M. D., Secretary, Knoxville.

Correspondents of the Society are requested to note that the address of the Secretary, Dr. C. C. Fite, has been changed from Nashville, Tenn., to *Knoxville, Tenn.* The Library of the Society will remain in the office of the State Board of Health, in the Capitol at Nashville, but all communications and exchanges should be addressed, as above, to Knoxville.

